# Peptide Substrates for Rho-Associated Kinase 2 (Rho-Kinase 2/ROCK2)

**DOI:** 10.1371/journal.pone.0022699

**Published:** 2011-07-27

**Authors:** Jeong-Hun Kang, Daisuke Asai, Akira Tsuchiya, Takeshi Mori, Takuro Niidome, Yoshiki Katayama

**Affiliations:** 1 Department of Biomedical Engineering, National Cerebral and Cardiovascular Center Research Institute, Suita, Osaka, Japan; 2 Department of Microbiology St. Marianna University School of Medicine, Kawasaki, Japan; 3 Department of Applied Chemistry, Faculty of Engineering, Kyushu University, Fukuoka, Japan; 4 Center for Future Chemistry, Kyushu University, Fukuoka, Japan; University Paris Diderot-Paris 7, France

## Abstract

Peptide substrates sensitive for a certain protein kinase could be important for new-drug development and to understand the mechanism of diseases. Rho-associated kinase (Rho-kinase/ROCK) is a serine/threonine kinase, and plays an important part in cardiovascular disease, migration and invasion of tumor cells, and in neurological disorders. The purpose of this study was to find substrates with high affinity and sensitivity for ROCK2. We synthesized 136 peptide substrates from protein substrates for ROCK2 with different lengths and charged peptides. Incorporation of ^32^P [counts per minute (CPM)] for each peptide substrate was determined by the radiolabel assay using [γ-^32^P]ATP. When the top five peptide substrates showing high CPMs (R4, R22, R133, R134, and R135) were phosphorylated by other enzymes (PKA, PKCα, and ERK1), R22, R133, and R135 displayed the highest CPM level for ROCK2 compared with other enzymes, whereas R4 and R134 showed similar CPM levels for ROCK2 and PKCα. We hypothesize that R22, R133, and R135 can be useful peptide substrates for ROCK2.

## Introduction

Phosphorylation by protein kinases plays an essential part in the signal transduction pathways that regulate cellular functions in response to extracellular signals. It is also a general mechanism for the control of intracellular processes [1]. Designing specific peptide substrates for a certain protein kinase is indispensable for the characterization of (or search for) substrate proteins for enzymes, and is also important in explorations for new drugs. In general, peptide substrates specific to a certain protein kinase are identified using genetic variants of protein substrates and synthetic peptides [2]–[4].

Rho-associated kinase (Rho-kinase/ROCK; hereafter referred to as ROCK) is a serine/threonine kinase and plays an important part in various cellular functions. These include the contraction of smooth muscle, cell adhesion, and cytokinesis [5], [6]. Over-expression of this enzyme has been associated with cardiovascular diseases such as hypertension and cerebral and coronary vasospasm [7]–[9]. Moreover, ROCK is closely associated with the migration and invasion of tumor cells [10], [11]. Recent studies suggest that inhibition of ROCK not only increases cerebral blood flow and leads to protection against stroke, but enhances functional recovery in injured spinal cords [12]–[14]. Thus, ROCK could be a potential therapeutic target for tumors, neurological disorders and cardiovascular diseases.

ROCK is divided into two isozymes: ROCK1 (ROCKβ) and ROCK2 (ROCKα) [5], [6]. The latter is mainly expressed in the brain, heart, and skeletal muscle, and ROCK1 has been identified in the spleen, lung, liver, testis, and kidney [15]. Many protein substrates for ROCK and their substrate sequences have been reported. ROCK can phosphorylate calponin [16]; LIM-kinase 1 (LIMK1) [17]; adducin [18]; intermediate filaments (vimentin [19], neurofilament-L [20], and glial fibrillary acidic protein (GFAP) [21]); collapsin response mediator protein 2 (CRMP2) [22]; tau [23]; microtubule-associated protein 2 (MAP2) [23]; ERM family (ezrin/radixin/moesin) [24]; myosin binding subunit (MBS) [25]; myosin light-chain (MLC) [26]; myristoylated alanine-rich C kinase substrate (MARCKS) [27], [28]; Rho E [29]; zipper-interacting protein kinase (ZIPK) [30]; LIMK2 [31] and endophilin A1 [32].

The purpose of the present study was to find substrates with high affinity and sensitivity for ROCK2. We synthesized 136 peptide substrates from protein substrates for ROCK2 with different lengths and charged peptides. After determining the incorporation of radioactivity (counts per minute (CPM)) for each peptide substrate by the radiolabel assay using [γ-^32^P]ATP, kinetic properties for the final five top-ranked substrates were analyzed. We also examined the affinities of five substrates for other kinases (extracellular signal-regulated kinase 1 (ERK1), protein kinase A (PKA), and protein kinase C (PKC)α) showing similar consensus sequences and interactive functions in intracellular signal transduction to ROCKs.

## Results

To search for peptide substrates with high affinity and sensitivity for ROCK2, we synthesized 136 peptide substrates on the basis of the amino-acid sequences in ROCK protein substrates (Table S1 and Table S2). These peptide substrates showed a different number of charged peptide residues or amino-acid residues. Peptide substrates had a basic amino acid (Arg or Lys) at amino-terminal position −2 or −3 of the phosphorylated sites (Ser and Thr). Moreover, most amino acids at the +2 carboxyl-terminal position were basic or hydrophobic (Arg, Lys, Phe, Leu, Trp, or Val) (Table S2).

**Figure 1:**
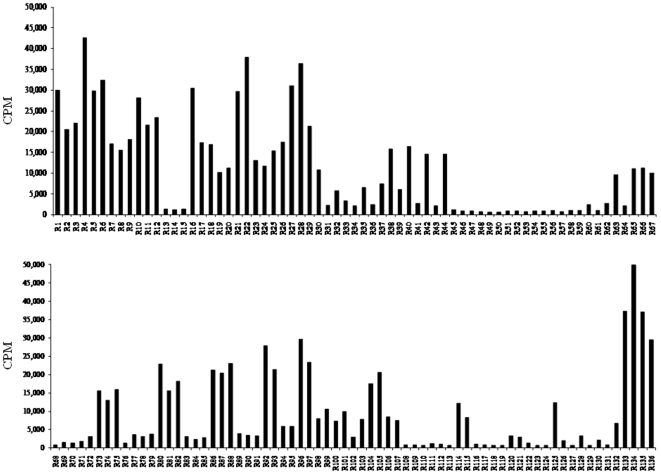
Counts per minute (CPM) levels for 136 peptide substrates. Each peptide substrate was analyzed by the radiolabel assay using [γ-^32^P]ATP.

**Figure 2:**
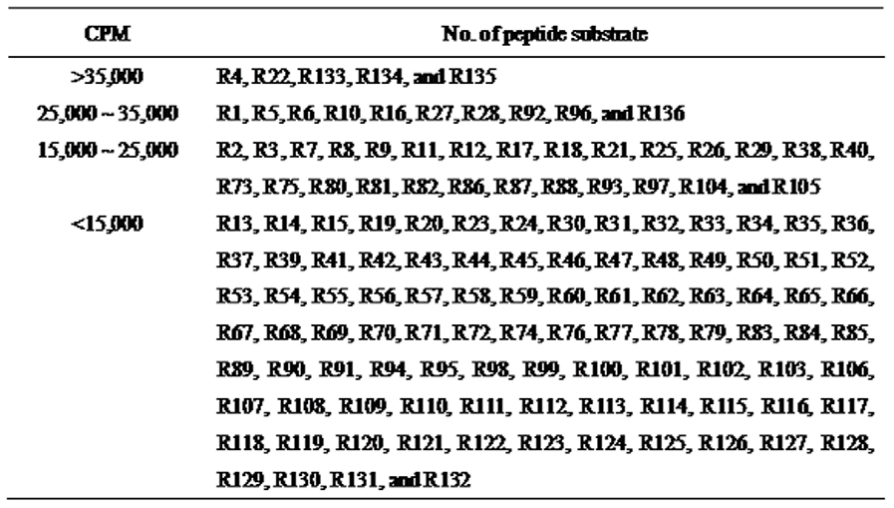
Classification of peptide substrates. Four groups were divided from the results of radioactivity (*i.e.*, CPM).

We determined the radioactivity (in counts per minute (CPM)) for each peptide substrate by the radiolabel assay using [γ-^32^P]ATP (Fig. 1). Peptide substrates were divided into four groups from the results of radioactivity. Conversely, five peptide substrates showing CPM values of >35,000 for ROCK2 were in the order R134 (KSARKKRYTVVGNPYWM)>R4 (RAKYKTLRQIR)>R22 (KPARKKRYTVVGNPYWM)>R133 (KSDRKKRYTVVGNPYWM)>R135 (KADRKKRYTVVGNPYWM) (Fig. 2).

The Ser∶Thr ratio for the total number of phosphorylation sites used in this study was 9∶12. The substitution of Ser for Thr at the phosphorylation site led to a tendency to a decrease in the radioactivity (see R1–6 and R7–12, R64–67 and R68–71, R72–75 and R76–79, R80–82 and R83–85, and R86–88 and R89–91) (Fig. 1). Although the observation was not universal, the change of negatively charged amino acids (Asp or Glu) into Ala or positively charged amino acids tended to result in an increase in CPM. However, the length of peptides had no or very little effect on CPMs (Fig. 1 and Table S2).

Furthermore, the kinetic properties of five peptide substrates showing high CPM for ROCK2 were analyzed. Values of *K_m_* and *V_max_* for five peptide substrates ranged from 1.7 µM to 3.3 µM, and from 8.6 pmol/min/mg to 14.9 pmol/min/mg, respectively (Table 1).

**Table 1:** Kinetic properties for five peptide substrates showing CPM>35,000.

Number of peptide substrate	*K_m_* (µM)	*V_max_* (pmol/min/mg)	*V_max_/K_m_*
R4	1.7	14.2	8.4
R22	1.9	10.3	5.4
R133	3.6	10.0	2.8
R134	3.3	14.9	4.5
R135	3.2	8.6	2.7

**Figure 3:**
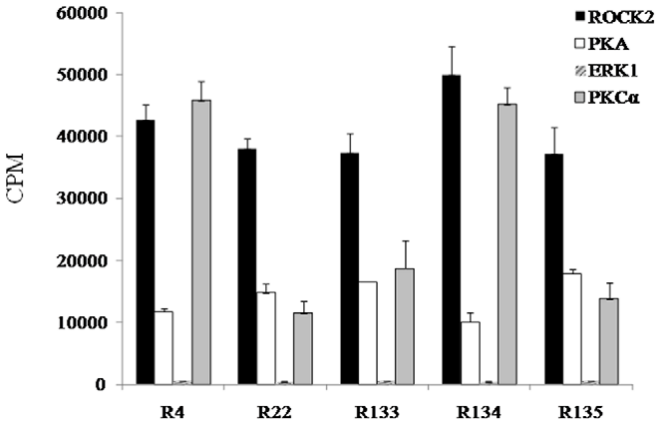
Phosphorylation of the top five peptide substrates (R4, R22, R133, R134, and R135) for ROCK2 by other enzymes (PKA, PKCα, or ERK1). Each peptide substrate was analyzed by the radiolabel assay using [γ-^32^P]ATP. CPM, counts per minute.

The consensus sequence of the ROCK2 phosphorylation site was considered to be R/KXXS/T or R/KXS/T (R, arginine; K, lysine; X, any amino acid; S, serine; and T, threonine) [5], [33] (Table S2). These consensus sequences were similar to other protein kinases (e.g., PKA and PKC). PKA recognizes the phosphorylation motifs R/KXS/T and R/KXXS/T [34], [35]. Consensus phosphorylation site motifs for PKC were identified to be R/KXXS/T, R/KXS/T, R/KXXS/TXR/K, or R/KXS/TXR/K [35], [36]. Mitogen-activated protein kinase (MAPK) can recognize the phosphorylation motif XS/TPX (P, proline) [37]. Five peptide substrates that showed CPM of >35,000 for ROCK2 were phosphorylated with PKA, PKCα (isozyme of PKC), and ERK1 (isozyme of MAPK). R4 and R134 showed high CPM for ROCK2 and PKCα. R22, R133, and R135 showed much higher CPM for ROCK2 than that of PKCα (Fig. 3).

Several studies have reported that replacement of phosphorylatable amino acids with alanine can lead to an inhibitor peptide for each enzyme [38], [39]. Thus, we examined if peptides that replace phosphorylatable amino acids with alanine can inhibit the phosphorylation of substrate peptide by ROCK2. The phosphorylatable threonine of five peptide substrates (R4, R22, R133, R134, and R135) was replaced with alanine and used as inhibitor peptides. The lowest *K_i_* (11.6 µM) in a competitive manner was obtained for R22 with alanine instead of threonine (Table 2).

**Table 2:** *K_i_* values for five peptides with alanine instead of a phosphorylatable threonine.

Number of peptide	*K_i_* (µM)
R4	86.6
R22	11.6
R133	>100
R134	29.2
R135	>100

## Discussion

There have been many efforts to search for peptide substrates with high affinity and sensitivity to ROCK2. However, different experimental methods make selecting substrates for ROCK2 quite difficult. Moreover, the affinity and sensitivity of substrates for ROCK2 can be influenced by several factors. These include the peptide length and the number of charged peptides [38], [40]–[42], but these factors were not investigated in previous studies.

We synthesized 136 peptide substrates for ROCK2 with different lengths and charged peptides, and analyzed their ability for phosphorylation. When negatively charged amino acids (Asp and Glu) were replaced by Ala or a positively charged amino acid (Arg), resulting in an increase in the positive charge density in peptide substrate, a tendency to an increase in the radioactivity was identified. For example, a higher CPM value was identified for R4 than for R1, R38 than R33, and R134 than R133. The length of peptide substrate had no or little effect on the level of radioactivity. In general, shortening of peptide substrates decreased the affinity for the enzyme (*i.e.*, increased the *K_m_* value). However, the peptide length showed minor changes in the *V_max_* value [38], [40], [41]. These data showed that radioactivity was not affected by the length of peptide substrates, but that the change of negatively charged amino acids into Ala or positively charged amino acids could increase the radioactivity values.

PKA and PKCα broadly recognize ROCK phosphorylation sites because of their similar consensus sequences to the ROCK phosphorylation motif. Our previous study showed that ROCK substrates can be good substrates for PKA and/or PKCα. Among 18 ROCK phosphorylation sites, 14 sites (Thr-184 of calponin; Thr-508 of LIMK1; Ser-71 of vimentin; Ser-57 of neurofilament-L; Thr-7, Ser-13, and Ser-34 of GFAP; Thr-555 of CRMP2; Ser-409 of tau; Ser-1796 of MAP2; Ser-854 of MBS; Thr567/564/558 of the ERM family; Ser-19 of MLC; and Ser-159 of MARCKS) were phosphorylated by PKA and/or PKCα [33]. Moreover, there was a close function in intracellular signal transduction between ROCK and PKCα, ERK1, or PKA. The interactive role of ROCK and PKCα has been reported in the actin–myosin interaction [43] and in the contraction of vascular smooth muscle [44], [45]. MLC phosphorylation was stimulated by ROCK and PKCα, but decreased by suppressing ROCK and PKCα using inhibitors [44], [45]. RhoA phosphorylation at Ser 188 by PKA blocks the ROCK pathways, resulting in activation of MLC phosphatase and the relaxation of smooth muscle [46]. Activated PKA can also facilitate axon formation by inhibiting RhoA, but ROCK has opposite effects on axon formation [47]. ROCK has an effect on translocation of ERK1 to the cellular nucleus, leading to proliferation of smooth muscle cells in the pulmonary artery [48]. ERK1 activation by ROCK stimulates: 1) force-induced osteopontin expression in human periodontal ligament fibroblasts through focal adhesion kinase signaling [49]; and 2) migration and proliferation of glioblastoma cells [50]. The observations noted above mean that PKA, PKCα, and ERK1 directly and/or indirectly participate in the signal transduction pathways of ROCK. Thus, we examined if five peptide substrates (R4, R22, R133, R134, and R135) showing high CPM values (>35,000) for ROCK2 could be phosphorylated by other enzymes (PKA, PKCα, or ERK1). R22, R133, and R135 displayed higher CPM levels for ROCK2 than the other enzymes, whereas R4 and R134 showed similar CPM levels for ROCK2 and PKCα (Fig. 3). These results suggested that R22 and R133 could be useful peptide substrates for ROCK2, and that R4 and R134 could be useful peptide substrates for ROCK2 and PKCα.

Replacement of phosphorylation sites with alanine is used to develop inhibitor peptides. In general, peptides showing higher affinity (*i.e.*, low *K_m_*) than lower affinity (i.e., high *K_m_*) become much more effective inhibitor peptides when phosphorylation sites are substituted by alanine. In the present study, we examined if five peptides in which a phosphorylatable amino acid (threonine) was replaced with alanine could be used as potent competitive inhibitors of ROCK2. The R22 peptide with alanine instead of threonine showed the lowest *K_i_* (11.6 µM) (Table 2).

In conclusion, there has been an increasing interest in ROCKs as therapeutic targets of cardiovascular disease, migration and invasion of tumor cells, and neurological disorders. Peptide substrates with high affinity and sensitivity to ROCK2 are important in new-drug developments and in understanding the cellular signals involved. In the present study, 136 peptide substrates from protein substrates for ROCK2 were synthesized and the radioactivity of each peptide substrate analyzed. From CPM results and phosphorylation reactions with other enzymes (PKA, PKCα, and ERK1), we found three peptides (R22, R133, and R135) to be useful peptide substrates for ROCK2.

## Materials and Methods

### Syntheses of peptide substrates

Peptide substrates were synthesized using an automatic peptide synthesizer according to standard Fmoc-chemistry procedures. After treatment with trifluoroacetic acid (TFA), peptides were purified on an Inertsil ODS-3 column (250×20 mm, 3.5 µm; GL Sciences, Tokyo, Japan) using a BioCAD Perfusion Chromatography System (Ikemoto Scientific Technology, Tokyo, Japan) and a linear A–B gradient at a flow rate of 8 mL/min where eluent A was 0.1% TFA in water and eluent B was 0.1% TFA in acetonitrile. The purity of synthetic peptide was identified by high-performance liquid chromatography and matrix-assisted laser desorption/ionization-time-of-flight mass spectrometry, and the peptide with >95% purity was used for the phosphorylation reaction.

### Phosphorylation of peptide substrates by enzymes

The synthetic peptide S6K (KRRRLASLR) was used as a control for ROCK2. Alphatomega (FKKQGSFKKK) [4] was used for PKCα assays and ZIPK-T265 (KRRMTIAQSLEHSWIK) [30] for PKA assays. ERK assays employed 0.33 mg/mL human myelin basic protein (MBP; Chemicon International, Temecula, CA, USA).

The kinase activity of recombinant ROCK2 (Carna Biosciences, Kobe, Japan), ERK1 (Upstate, Millipore, Temecula, CA, USA), PKA (Promega, Madison, WI, USA), and PKCα (Sigma–Aldrich, St. Louis, MO, USA) for the peptide substrates was determined by measuring ^32^P transfer from [γ-^32^P]ATP into substrate peptides according to manufacturer recommendations. Phosphorylation reactions by ROCK2 were carried out in 25 µL of buffer (5 mM 3-(N-morpholino)propanesulfonic acid (MOPS) at pH 7.2, 2.5 mM beta-glycerophosphate, 1 mM ethylene glycol tetra-acetic acid (EGTA), 4 mM MgCl_2_, 0.05 mM dithiothreitol (DTT), and 100 µM adenosine triphosphate (ATP)) containing peptides and 1.25 µg/ml ROCK2. For PKCα, phosphorylation reactions were carried out in 25 µL of buffer (20 mM HEPES at pH 7.4, 10 mM MgCl_2_, 1 mM CaCl_2_, 100 µM ATP, 40 µg/mL phosphatidylserine, and 20 µg/mL diacylglycerol) containing peptides and 0.5 µg/mL PKCα. Phosphorylation reactions by PKA were carried out in 25 µL of buffer (50 mM HEPES at pH 7.4, 1 mM CaCl_2_, 10 mM MgCl_2_, 100 µM ATP) containing peptides and 1.6 µg/mL PKA. For ERK1, phosphorylation reactions were carried out in 25 µL of buffer (25 mM Tris-HCl at pH 7.5, 20 µM EGTA, 15 mM magnesium acetate, and 100 µM ATP) containing peptides and 0.5 µg/mL ERK1. For each experimental condition, values for control reactions lacking substrate peptides were subtracted as blanks. The assay mixture was incubated for 10 min at 25°C, and the reaction terminated by the addition of 5 µL of 30% trichloroacetic acid (TCA). The reaction mixture (24 µL) was spotted onto P-81 phosphocellulose membranes. The membranes were washed thrice with 5% TCA, dried with acetone, and the radioactivity of each membrane determined by liquid scintillation counting. Kinetic analyses of enzyme activity were determined using the Lineweaver–Burk plot.

### Inhibition of phosphorylation by inhibitor peptides

The phosphorylatable amino acid (threonine) of five peptide substrates (R4, R22, R133, R134, and R135) was replaced with alanine. Inhibitory activity of these inhibitor peptides against ROCK2 was examined using S6K peptide (KRRRLASLR) as a substrate; value of *K_m_* and *V_max_* for S6K was 3.1 µM and 23.1 pmol/min/mg, respectively. *K_i_* values were obtained from the Dixon plot and Lineweaver–Burk plot. Inhibitor peptides were added into 25 µL of buffer (5 mM MOPS at pH 7.2, 2.5 mM beta-glycerophosphate, 1 mM EGTA, 4 mM MgCl_2_, 0.05 mM DTT, and 100 µM ATP) containing S6K substrate peptide and 1.25 µg/mL ROCK2.

## Supporting Information

Table S1 **Origins of each peptide substrate for ROCK2.**
(DOCX)

Table S2 **Sequences of peptide substrate for ROCK2.**
(DOCX)
