# Supplementary material for: Peptide Substrates for Rho-Associated Kinase 2 (Rho-Kinase 2/ROCK2)
Source: PLoS One. 2011 Jul 27;6(7):e22699. doi: 10.1371/journal.pone.0022699 (PMC3144920; doi:10.1371/journal.pone.0022699)
Supplement: Table S1 — Origins of each peptide substrate for ROCK2. (DOCX) [file pone.0022699.s001.docx]

Table S1. Origins of each peptide substrate for ROCK2

| Origin | No. of peptide substrate |
| --- | --- |
| ERM family (Ezrin/ Radixin/ Moesin) | R1 ~ R12 |
| Vimentin | R13 ~ R15 |
| LIM-kinase 1 (LIMK1) | R16 ~ R28 |
| Myristoylated alanine-rich C kinase substrate (MARCKS) | R29 and R30 |
| Collapsin response mediator protein 2 (CRMP2) | R31 ~ R40 |
| Glial fibrillary acidic protein (GFAP) | R41 ~ R44 |
| GFAP | R45 and R46 |
| Neurofilamen-L | R47 and R48 |
| Calponin | R49 ~ R54 |
| Calponin | R55 ~ R58 |
| Myosin light chain (MLC) | R59 ~ R63 |
| Adducin | R64 ~ R91 |
| Myosin binding subunit (MBS) | R92 ~ R107 |
| Microtubule associated protein 2 (MAP2) | R108 and R109 |
| Tau | R110 and R111 |
| Tau | R112 ~ R115 |
| Tau | R116 ~ R121 |
| RhoE | R122 ~ R124 |
| Endophilin A1 | R125 ~ R132 |
| LIMK2 | R133 ~ R135 |
| Zipper-interacting protein kinase (ZIPK) | R136 |
