# Supplementary material for: Peptide Substrates for Rho-Associated Kinase 2 (Rho-Kinase 2/ROCK2)
Source: PLoS One. 2011 Jul 27;6(7):e22699. doi: 10.1371/journal.pone.0022699 (PMC3144920; doi:10.1371/journal.pone.0022699)
Supplement: Table S2 — Sequences of peptide substrate for ROCK2. (DOCX) [file pone.0022699.s002.docx]

Table S2. Sequences of peptide substrate for ROCK2 (*continued*)

(*Continued on next page*)

Table S2. Sequences of peptide substrate for ROCK2 (*continued*)

^a^ The number zero (gray) is the phosphorylation site of peptide substrate.
